# Less airway inflammation and goblet cell metaplasia in an IL-33-induced asthma model of leptin-deficient obese mice

**DOI:** 10.1186/s12931-021-01763-3

**Published:** 2021-06-01

**Authors:** Atsushi Kurokawa, Mitsuko Kondo, Ken Arimura, Shigeru Ashino, Etsuko Tagaya

**Affiliations:** 1 Department of Respiratory Medicine, Tokyo Women’s Medical University, Tokyo, 162-8666 Japan; 2 Department of Microbiology and Immunology, Tokyo Women’s Medical University, Tokyo, 162-8666 Japan

**Keywords:** Airway hyperresponsiveness, Asthma, Eosinophils, Goblet cell metaplasia, IL-33, Innate immunity, Leptin, Obesity

## Abstract

**Background:**

Obesity-associated asthma is a phenotype of severe asthma. Late-onset, non-eosinophilic and female-dominant phenotype is highly symptomatic and difficult to treat. Leptin, an adipokine, exerts an immunomodulatory effect. IL-33 associated with innate immunity induces type 2 inflammation and is present in adipose tissue. The purpose of this study was to elucidate the pathogenesis of obesity-associated asthma by focusing on the interaction between leptin and IL-33.

**Methods:**

In leptin-deficient obese (ob/ob) and wild-type mice, IL-33 was instilled intranasally on three consecutive days. In part of the mice, leptin was injected intraperitoneally prior to IL-33 treatment. The mice were challenged with methacholine, and airway hyperresponsiveness (AHR) was assessed by resistance (Rrs) and elastance (Ers) of the respiratory system using the forced oscillation technique. Cell differentiation, IL-5, IL-13, eotaxin, keratinocyte-derived chemokine (KC) in bronchoalveolar lavage fluid (BALF) and histology of the lung were analyzed. For the in vitro study, NCI-H292 cells were stimulated with IL-33 in the presence or absence of leptin. Mucin-5AC (MUC5AC) levels were measured using an enzyme-linked immunosorbent assay.

**Results:**

Ob/ob mice showed greater Rrs and Ers than wild-type mice. IL-33 with leptin, but not IL-33 alone, enhanced Ers rather than Rrs challenged with methacholine in ob/ob mice, whereas it enhanced Rrs alone in wild-type mice. IL-33-induced eosinophil numbers, cytokine levels in BALF, eosinophilic infiltration around the bronchi, and goblet cell metaplasia were less in ob/ob mice than in wild-type mice. However, leptin pretreatment attenuated these changes in ob/ob mice. MUC5AC levels were increased by co-stimulation with IL-33 and leptin in vitro.

**Conclusions:**

Ob/ob mice show innate AHR. IL-33 with leptin, but not IL-33 alone, induces airway inflammation and goblet cell metaplasia and enhances AHR involving peripheral airway closure. This is presumably accelerated by mucus in ob/ob mice. These results may explain some aspects of the pathogenesis of obesity-associated asthma.

## Background

The incidence of obesity is on the rise worldwide and is currently a critical public health issue. Obesity is a risk factor for the development of asthma and is associated with poor control and frequent exacerbations. Obese individuals with asthma have more severe symptoms, a lower quality of life, and an attenuated response to medication [1]. Obesity-associated asthma is a complex syndrome, including various phenotypes of the disease [2]. One is the early onset and typically atopic phenotype (higher IgE levels) [3]. Therefore, anti-inflammatory intervention and weight loss are potential therapies for this condition. The other phenotype represents late-onset, non-eosinophilic, and female-dominant phenotype with intense symptoms [4]. There are few specific treatments for this condition except for weight loss [5]. Various approaches, including lung function and adipokines such as leptin, have been used to understand the pathogenesis of obesity-associated asthma.

Obesity physiologically shows restrictive impairment due to excessive abdominal fat and low compliance of the thorax. Obesity reduces functional residual capacity (FRC), attenuates the tethering force between the airway and parenchyma, and induces the closure of peripheral airways [2]. Consequently, obesity-associated asthma may become more severe than other types of asthma.

Leptin is a hormone secreted by adipocytes and acts on the hypothalamus to inhibit hunger and stimulate satiety. In obesity, serum leptin levels are generally elevated because leptin resistance occurs and the feeling of hunger continues despite high energy stores [6]. Leptin is known to modulate innate and adaptive immune responses [7–10]. It is thought to be involved in the pathogenesis of obesity-associated asthma. In fact, leptin enhances airway responsiveness in ovalbumin (OVA)-sensitized mice [11]. OVA-sensitized leptin-deficient obese mice (ob/ob mice) show enhanced airway hyperresponsiveness (AHR) without an increase in type-2 inflammation [12]. However, the role of leptin in the innate immunity-related asthma model has not yet been fully investigated.

IL-33 is associated with innate immunity and induces type-2 inflammation in the airway. IL-33 is released from the injured airway epithelial cells and stimulates type-2 innate lymphocytes (ILC2) which release IL-5 and IL-13. These cytokines lead to eosinophilic inflammation, goblet cell metaplasia, and hyperresponsiveness in the airway [13]. IL-33 and its receptor ST2 have also been shown to be present in human adipose tissue [14]. It has recently been reported that circulating levels of IL-33 are elevated by obesity [15]. However, the role of IL-33-induced type 2 inflammation in obesity-associated asthma has not yet been established.

We asked whether IL-33 and leptin, both expressed in the airway [16, 17], might interact with each other and contribute to the pathogenesis of obesity-associated asthma. To simplify the role of leptin in the IL-33-induced asthma model, we used leptin-deficient ob/ob mice. We then investigated the effect of exogenous leptin treatment. Thus, we examined the effect of IL-33 on eosinophilic inflammation, goblet cell metaplasia, and airway responsiveness in ob/ob mice and wild-type C57BL/6 J mice. We assessed how exogenous leptin influences the IL-33-induced asthma model. Furthermore, we focused on the importance of mucus in in vivo and in vitro studies using NCI-H292 cells.

## Methods

### Animal models

**Fig. 1:**
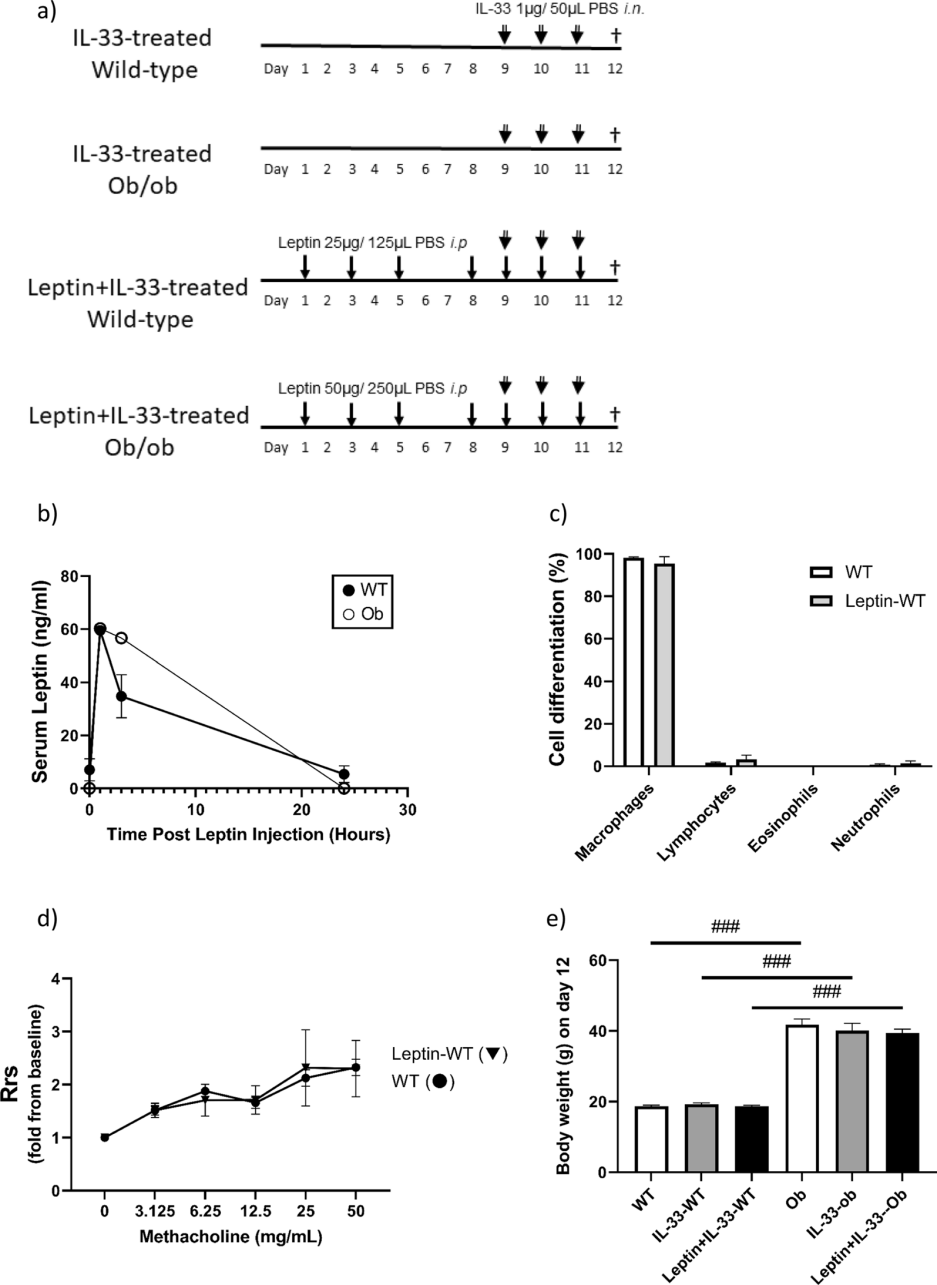
**a** Experimental protocol. In the leptin-treated groups, leptin (25 μg/125 μL phosphate-buffered saline [PBS] for wild-type, 50 μg/250 μL PBS for ob/ob) was injected intraperitoneally on days 1, 3, 5, and 8–11. In the IL-33-treated groups, IL-33 (1 μg/50 μL PBS) was instilled intranasally on days 9–11. On day 12, airway responsiveness, bronchoalveolar lavage fluid (BALF), and lung sections were assessed. **b** Effect of exogenous leptin on endogenous serum leptin concentrations over time. The mice were given a single intraperitoneal injection of leptin (25 μg for wild-type, 50 μg for ob/ob). Serum leptin levels were measured at 0, 1, 3 and 24 h after injection. Data are expressed as mean ± standard error of the mean (SEM). n = 2–3 for each group. **c** The cell differentials of BALF and **d** airway responsiveness to methacholine in leptin-treated wild-type mice. Resistance of the respiratory system (Rrs). n = 4 for each group. **e** Body weight on day 12. Data are expressed as mean ± standard error of the mean (SEM). n = 7–9 for each group. ^###^*p* < 0.001 vs. mice with an identical treatment

The animal protocol was approved by the Animal Care and Use Committee of Tokyo Women’s Medical University (license number: AE20-065-B). Ob/ob mice (genetically leptin-deficient obese mice, female, 7–9 weeks old, Charles River, Yokohama, Japan) and C57BL/6 J wild-type mice (age and sex-matched with ob/ob mice, Japan SLC, Hamamatsu, Japan) were divided into three groups (non-treated, IL-33-treated, and Leptin + IL-33-treated). Recombinant mouse IL-33 (SRP3210, Sigma-Aldrich, St Louis, MO, USA: 1 μg dissolved in 50 μL phosphate-buffered saline [PBS]) was instilled intranasally on days 9–11. Recombinant murine leptin (450–31, PeproTech, Cranbury, NJ, USA: 25 μg/125 μL PBS for wild-type, 50 μg/250 μL PBS for ob/ob) was injected intraperitoneally on days 1, 3, 5, and 8–11. This was according to the experimental protocol shown in Fig. 1a. As preliminary experiments, wild-type mice treated with leptin alone (on days 1, 3, 5, and 8–11) were also included in this study. To verify that exogenous i.p. treatment with leptin induced elevated serum levels of leptin, we treated the mice with a single exogenous injection of leptin (25 μg for wild-type, 50 μg for ob/ob). We then monitored their serum leptin levels at 0, 1, 3, and 24 h after injection. Serum levels of leptin were measured using an enzyme-linked immunosorbent assay (ELISA) kit (RD291001200R, BioVendor, Brno, Czech Republic). In addition, as a separate experiment, the baseline levels of IL-33 in BALF were confirmed by ELISA (ab213475, Abcam, Cambridge, United Kingdom) in non-treated ob/ob mice and in non-treated wild-type mice. The samples were run in duplicate. The limits of detection of ELISA were 30 pg/ml for leptin, 5.3 pg/ml for IL-33.

### Measurement of airway responsiveness

Mice were anesthetized by an intraperitoneal injection of pentobarbital (75 mg/kg) and zylazine (10 mg/kg) and ventilated (flexiVent; SCIREQ, Montreal, Canada). They were challenged with increasing doses of inhaled methacholine (3.125, 6.25, 12.5, 25, and 50 mg/ml, purchased from Sigma-Aldrich, St Louis, MO, USA). The resistance of the respiratory system (Rrs), elastance (Ers), Newtonian resistance (Rn), tissue damping (G), and tissue elastance (H) were measured using the forced oscillation technique as previously described [18]. Baseline Rrs, Ers, Rn, G, and H before methacholine inhalation were compared between wild-type and ob/ob mice. Airway responsiveness was assessed by a fold-change from the baseline.

### Bronchoalveolar lavage fluid (BALF) analysis

After measuring airway responsiveness, the BALF was collected by lavaging the lungs with 1.8 ml PBS. The BALF was centrifuged at 500×*g* for 3 min, and the supernatant was collected for subsequent analysis. The total cell numbers were counted using a hemocytometer. The cell differentials were counted by staining the cells with May-Giemsa. Cytokine and chemokine levels (IL-5, IL-13, eotaxin, and keratinocyte-derived chemokine [KC]) were analyzed using a mouse ELISA kit. The samples were run in duplicate. The limits of detection were 1.0 pg/ml for IL-5 (Thermo scientific, Frederick, MD, USA), 1.5 pg/ml for IL-13, 3.0 pg/ml for eotaxin, and 2.0 pg/ml for KC (R&D system, Minneapolis, MN, USA).

### Lung histology

The lungs were fixed with 10% formalin and embedded in paraffin. Sections were cut 5 μm thick and stained with hematoxylin–eosin (HE), periodic acid-Schiff/Alcian-blue (PAS/AB), and Masson’s trichrome.

### Mucus score

To assess goblet cell metaplasia in the bronchi stained with PAS/AB, mucus scores were obtained as previously described [19]. In brief, bronchi with an internal diameter measuring > 200 μm in cross section were assessed. Scores were obtained based on the ratio of the goblet cell area to the whole cross-sectional epithelial area in each round bronchus. A score of 0 indicated none, a score of 1 indicated occupation of < 1/3 of the epithelial area, a score of 2 indicated occupation of ≥ 1/3 to < 2/3 of the epithelial area, and a score of 3 indicated occupation of ≥ 2/3 of the epithelial area. The mucus score was obtained by averaging the scores of the measured bronchi.

### In vitro study using NCI-H292 cells

For the in vitro study of mucin synthesis, the human pulmonary mucoepidermoid carcinoma cell line NCI-H292 cells were cultured in RPMI 1640 medium (GIBCO; Invitrogen Co. Grand Island, NY, USA) with 10% fetal calf serum (FCS), penicillin (100 U/ml), streptomycin (100 μg/ml), and fungizone (2.5 μg/ml) at 37 °C in a humidified 5% CO_2_ incubator. NCI-H292 cells were plated in 6-well culture dishes at an initial density of 3 × 10^5^/well. After confluence, the cells were cultured in the same medium with 0.5% FCS for 24 h. Then, the medium was replaced with serum-free medium and the cells were stimulated for 24 h with IL-33 (0.5 ng/ml), leptin (1 ng/ml), or IL-33 (0.5 ng/ml) + leptin (1 ng/ml). The concentrations of leptin and IL-33 were selected based on previous reports [20–22]. Mucin-5AC (MUC5AC) protein levels in cell lysates were measured using an ELISA kit (Cloud-Clone Corp, TX, USA) as previously described [19, 23]. Data are shown as percentages in the non-stimulated control cells.

### Statistical analysis

All data are expressed as the mean ± standard error of the mean (SEM). Statistical analyses were performed using the Prism 8 software package (GraphPad Software, San Diego, CA, USA). In the measurement of airway responsiveness, baseline parameters were evaluated by an unpaired t-test and airway responsiveness was evaluated by two-way repeated analysis of variance with Tukey’s post-hoc test. All other data were evaluated using one-way analysis of variance with Tukey’s post-hoc test. Statistical significance was set at *p* < 0.05.

## Results

### Preliminary experiments about effect of leptin i.p. alone and baseline IL-33

Using a single leptin injection, we observed an increase in serum leptin concentration over endogenous levels in wild-type mice. The leptin concentrations were nearly the same in ob/ob mice as in wild-type mice 1 h after injection (ob/ob 60.2 ± 0.9 ng/ml, wild-type 59.5 ± 1.2 ng/ml). Concentrations returned to baseline levels after 24 h (Fig. 1b). Leptin *i.p.* alone did not affect cell differentials in BALF (Fig. 1c), airway responsiveness (Rrs) (Fig. 1d), and histology (data not shown) in wild-type mice. Baseline IL-33 levels in BALF were not different between non-treated ob/ob mice and non-treated wild-type mice (ob/ob 86.6 ± 9.2 pg/ml, wild-type 85.4 ± 8.5 pg/ml, n = 6).

### Body weight

Body weight did not significantly change during the experiment in all mice. Ob/ob mice were significantly heavier than wild-type mice (ob/ob 41.77 ± 1.59 g, wild-type 18.71 ± 0.31 g; *p* < 0.001, on day 12) (Fig. 1e).

### Airway responsiveness

**Fig. 2:**
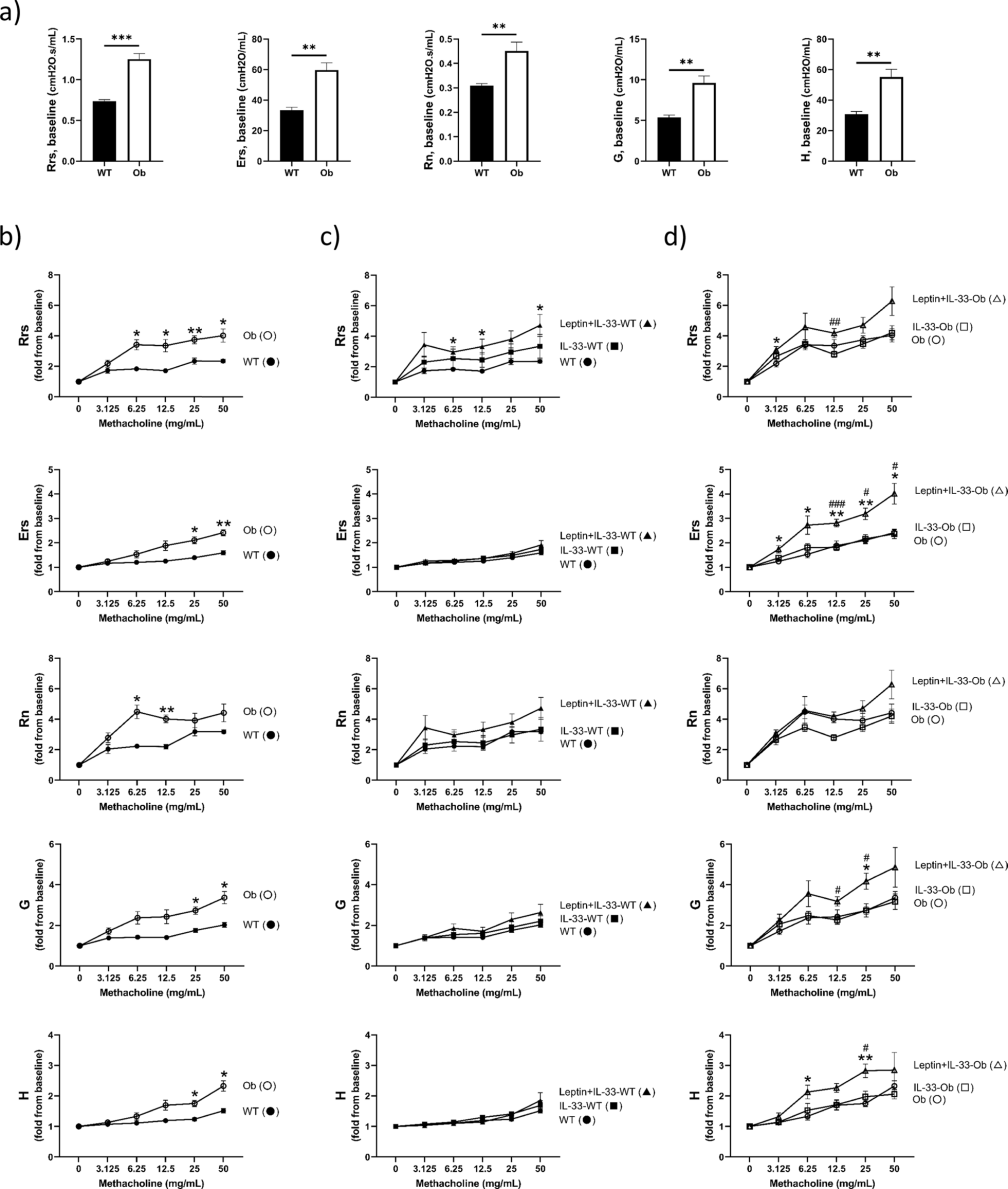
Airway responsiveness to inhaled methacholine. Resistance of the respiratory system (Rrs), elastance (Ers), Newtonian resistance (Rn), tissue damping (G), and tissue elastance (H). **a** Baseline parameters in wild-type and ob/ob mice. Airway responsiveness in **b** non-treated wild-type and ob/ob mice, **c** non-treated, IL-33-treated, Leptin + IL-33-treated wild-type mice, and **d** non-treated, IL-33-treated, Leptin + IL-33-treated ob/ob mice. Data are expressed as mean ± standard error of the mean (SEM). n = 5–9 for each group. **a** **p < 0.01, ***p < 0.001 vs. wild-type mice. **b**–**d** All parameters are shown as a fold-change from baseline. *Closed circle*: non-treated wild-type mice. *Open circle*: non-treated ob/ob mice. *Closed square*: IL-33-treated wild-type mice. *Open square*: IL-33-treated ob/ob mice. *Closed triangle*: Leptin + IL-33-treated wild-type mice. *Open triangle*: Leptin + IL-33-treated ob/ob mice. *p < 0.05, **p < 0.01 vs. non-treated. ^#^*p* < 0.05, ^##^*p* < 0.01, ^###^ vs. *p* < 0.001 vs. IL-33-treated

Ob/ob mice showed significantly greater baseline Rrs, Ers, Rn, G, and H than wild-type mice (Rrs: 1.253 ± 0.069 vs. 0.736 ± 0.021 cmH_2_O.s/ml; *p* < 0.001, Ers: 59.692 ± 4.744 vs. 33.419 ± 1.899 cmH_2_O/ml; *p* < 0.01, Rn: 0.451 ± 0.037 vs. 0.309 ± 0.009 cmH_2_O.s/ml; *p* < 0.01, G: 9.620 ± 0.842 vs. 5.378 ± 0.281 cmH_2_O/ml; *p* < 0.01, H: 55.078 ± 5.093 vs. 30.774 ± 1.767 cmH_2_O/ml; *p* < 0.01) (Fig. 2a). They also showed greater response to methacholine than wild-type mice (Fig. 2b). In ob/ob mice, Rrs, Ers, G, and H were significantly greater in Leptin + IL-33-treated mice than in non-treated mice at the concentrations of methacholine (Rrs; 3.125 mg/ml, Ers; all concentrations, G; 25 mg/ml, H; 6.25, and 25 mg/ml) (Fig. 2d). In wild-type mice, Rrs was significantly greater in Leptin + IL-33-treated mice than in non-treated mice at 6.25, 12.5, and 50 mg/ml of methacholine. Although, Ers, Rn, G, and H were not significantly changed (Fig. 2c).

### BALF analysis

**Fig. 3:**
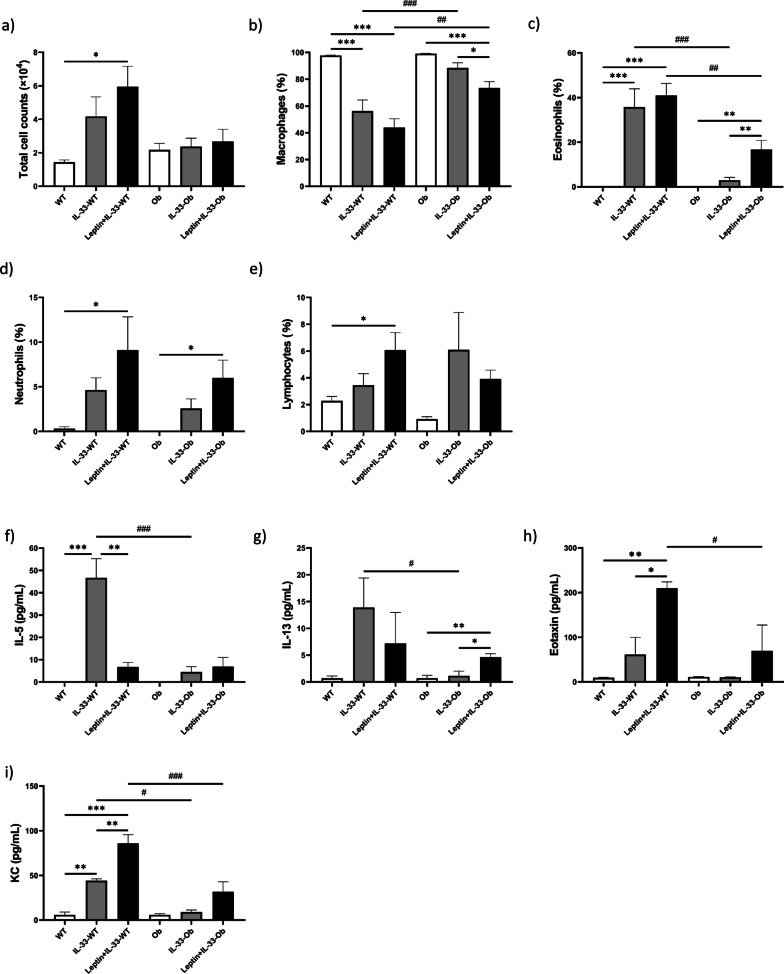
The cell differentials of bronchoalveolar lavage fluid. **a** Total cells, **b** % macrophages, **c** % eosinophils, **d** % neutrophils, and **e** % lymphocytes. The cytokine and chemokine analysis in bronchoalveolar lavage fluid. **f** IL-5, **g** IL-13, **h** Eotaxin, **i** KC (keratinocytes-derived chemokine). Data are expressed as mean ± standard error of the mean (SEM). **a**–**e** n = 10–12, **f**–**i** n = 6–9 for each group. *p < 0.05, **p < 0.01, *** *p* < 0.001 vs. genotype-matched mice. ^#^*p* < 0.05, ^##^*p* < 0.01, ^###^vs. *p* < 0.001 vs. mice with an identical treatment

The total cell counts in BALF were lower in IL-33-treated ob/ob mice than in IL-33-treated wild-type mice (2.37 ± 0.51 vs. 4.17 ± 1.17 × 10^4^ per ml) (Fig. 3a). In wild-type mice, IL-33 induced marked eosinophilia in BALF (non-treated vs. IL-33-treated: 0.0% vs. 35.7 ± 8.1%; *p* < 0.001). However, in ob/ob mice, IL-33 did not induce significant eosinophilia (non-treated vs. IL-33-treated: 0.0% vs. 2.9 ± 1.2%). In ob/ob mice, Leptin + IL-33 treatment induced a significant increase in eosinophils (IL-33-treated vs. Leptin + IL-33-treated: 2.9 ± 1.2% vs. 16.6 ± 4.1%; *p* < 0.01) (Fig. 3c). IL-5 and IL-13 levels in BALF were significantly lower in IL-33-treated ob/ob mice than in IL-33-treated wild-type mice (IL-5: 4.5 ± 2.4 vs. 46.6 ± 8.6 pg/ml; *p* < 0.001, IL-13: 1.1 ± 0.9 vs. 13.9 ± 5.5 pg/ml; *p* < 0.05) (Fig. 3f, g). Eotaxin levels tended to be lower in IL-33-treated ob/ob mice than in IL-33-treated wild-type mice (10.3 ± 0.5 vs. 61.5 ± 38.2 pg/ml) (Fig. 3h). KC levels were significantly lower in IL-33-treated ob/ob mice than in IL-33-treated wild-type mice (8.9 ± 2.3 vs. 44.1 ± 2.1 pg/ml; *p* < 0.05) (Fig. 3i). In ob/ob mice, Leptin + IL-33 treatment increased IL-5, IL-13, eotaxin, and KC levels (IL-33-treated vs. Leptin + IL-33-treated; IL-5: 4.5 ± 2.4 vs. 6.9 ± 4.2 pg/ml, IL-13: 1.1 ± 0.9 vs. 4.6 ± 0.6 pg/ml; *p* < 0.05, eotaxin: 10.3 ± 0.5 vs. 69.7 ± 57.7 pg/ml, KC: 8.9 ± 2.3 vs. 31.7 ± 11.1 pg/ml) (Fig. 3f–i). In wild-type mice, Leptin + IL-33 treatment significantly increased eotaxin and KC levels compared to IL-33 alone (IL-33-treated vs. Leptin + IL-33-treated; eotaxin: 61.5 ± 38.2 vs. 209.9 ± 13.9 pg/ml; *p* < 0.05; KC: 44.1 ± 2.1 vs. 85.9 ± 9.8 pg/ml; *p* < 0.01) (Fig. 3h, i).

### Histology

**Fig. 4:**
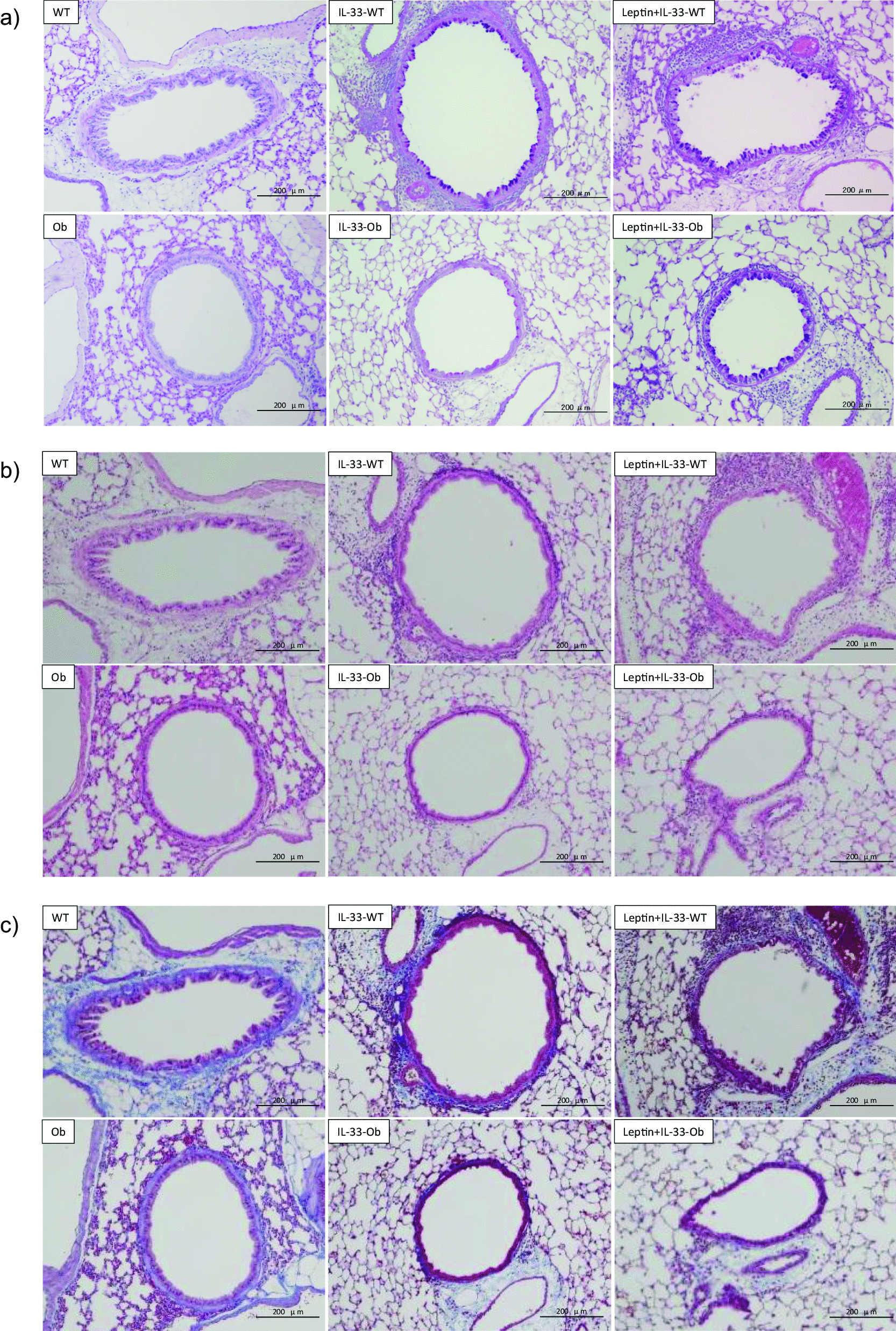
The representative light microscopic photographs of lung sections. **a** PAS/Alcian-blue, **b** hematoxylin–eosin, and **c** Masson’s trichrome stain. Upper panels; non-treated wild-type, IL-33-treated wild-type, Leptin + IL-33-treated wild-type, Lower panels; non-treated ob/ob, IL-33-treated ob/ob, Leptin + IL-33-treated ob/ob mice. Scale bar = 200 μm

In non-treated wild-type and ob/ob mice, neither airway inflammation nor goblet cell metaplasia were observed in PAS/AB and HE staining. In IL-33-treated wild-type mice, airway inflammation and goblet cell metaplasia were observed. In contrast, in IL-33-treated ob/ob mice, the changes were attenuated. However, the addition of exogenous leptin induced inflammation and goblet cell metaplasia (Fig. 4a, b). In Leptin + IL-33-treated wild-type mice, marked inflammation and goblet cell metaplasia were observed (Fig. 4a, b). Masson’s trichrome staining showed that peri-bronchial fibrous tissue shown by blue staining was less in ob/ob mice than in wild-type mice. A similar tendency was observed in IL-33 or IL-33 + Leptin-treated ob/ob mice (Fig. 4c).

### Mucus score

**Fig. 5:**
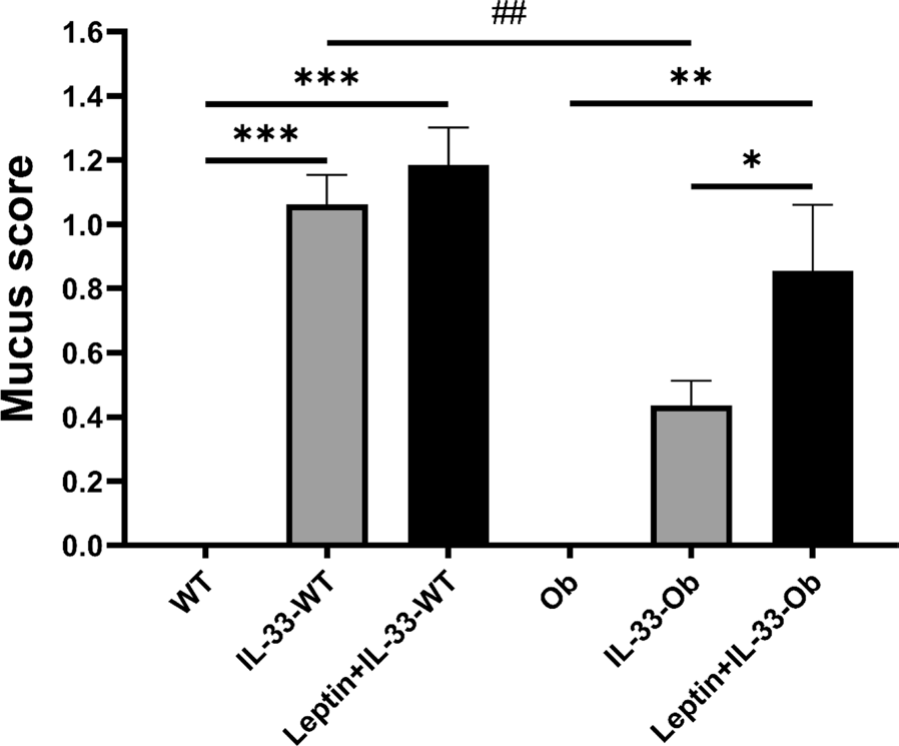
Mucus score. Data are expressed as mean ± standard error of the mean (SEM). n = 5–6 for each group. *p < 0.05, **p < 0.01, ***p < 0.001 vs. genotype-matched mice. ^##^*p* < 0.01 vs. mice with an identical treatment

The mucus score was lower in IL-33-treated ob/ob mice than in IL-33-treated wild-type mice (0.44 ± 0.08 vs. 1.06 ± 0.09; *p* < 0.01). However, the addition of exogenous leptin significantly increased the mucus score in ob/ob mice (IL-33-treated vs. Leptin + IL-33-treated; 0.44 ± 0.08 vs. 0.86 ± 0.21; *p* < 0.05) (Fig. 5).

### In vitro study using NCI-H292 cells

**Fig. 6:**
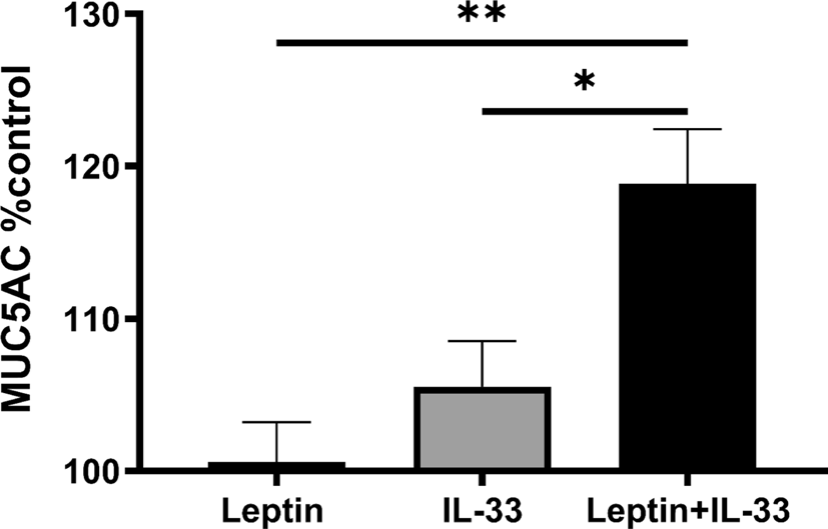
In vitro study using NCI-H292 cells. MUC5AC level induced by leptin and IL-33. Data are expressed as mean ± standard error of the mean (SEM). n = 5–6 for each group. *p < 0.05, Leptin + IL-33 vs. IL-33. **p < 0.01, Leptin + IL-33 vs. Leptin

MUC5AC levels did not change with leptin or IL-33 alone. However, they were increased by co-stimulation with leptin and IL-33 in vitro (Leptin vs. Leptin + IL-33; 100.6 ± 2.6% vs. 118.9 ± 3.6%; *p* < 0.01, IL-33 vs. Leptin + IL-33; 105.6 ± 3.0% vs. 118.9 ± 3.6%; *p* < 0.05) (Fig. 6).

## Discussion

In this study, ob/ob mice showed less eosinophilic inflammation and goblet cell metaplasia induced by IL-33 than wild-type mice. However, these changes were attenuated by exogenous leptin. Furthermore, AHR was elevated in ob/ob mice and IL-33 combined with leptin, however not IL-33 alone, enhanced the changes in Ers rather than Rrs induced by methacholine in ob/ob mice. In our in vitro study, the combination of leptin and IL-33 enhanced mucus production. These findings suggest that leptin enhances IL-33-induced eosinophilic inflammation and goblet cell metaplasia in the airway. Obesity per se may increase AHR without inflammation and the increase in mucus and inflammation by IL-33 combined with leptin enhanced AHR in obesity.

We demonstrated that IL-33-induced airway eosinophilic inflammation was attenuated in ob/ob mice (Fig. 3c). In addition, IL-5, IL-13, eotaxin, and KC levels in BALF were lower in IL-33-treated ob/ob mice than in IL-33-treated wild-type mice (Fig. 3f–i). Furthermore, the exogenous administration of leptin in ob/ob mice attenuated these changes. Classically, it is recognized that OVA-sensitized ob/ob mice do not show an increase in type-2 inflammation [12]. Given that IL-33 induces type-2 cytokines from ILC2, the attenuated eosinophilia in ob/ob mice may have been associated with a decrease in IL-5, IL-13, and eotaxin. Zheng et al. demonstrated that leptin promotes the proliferation of Th2 cells and ILC2s. They also demonstrated that leptin deficiency leads to reduced ILC2s and attenuated type-2 cytokine production [24]. Ding et al. reported that ILC2 was decreased in the adipose tissue of ob/ob mice [25]. Therefore, our results may have been caused by a decrease in ILC2 levels in ob/ob mice.

In wild-type mice, IL-33 induced goblet cell metaplasia (Fig. 4a) and increased the mucus score (Fig. 5). Conversely, in ob/ob mice, IL-33-induced goblet cell metaplasia was attenuated and exogenous leptin administration reversed this change (Fig. 4a). This may have been caused by the decrease in IL-13 in IL-33-treated ob/ob mice. This is because IL-13 plays an important role in the induction of goblet cell metaplasia [26]. Furthermore, in our in vitro study, MUC5AC levels were increased by co-stimulation with IL-33 and leptin (Fig. 6). Leptin and its receptors are expressed in the airway epithelium [17]. The asthmatic airway epithelium shows an increase in IL-33 expression [16]. Leptin has been reported to induce mucin protein expression in human airway epithelial cells [21]. Therefore, our in vitro study supports the hypothesis that leptin deficiency disturbs mucin production in the IL-33-stimulated airway epithelium. It also supports the results that exogenous leptin treatment induced mucin production in IL-33-treated ob/ob mice.

Subsequently, ob/ob mice spontaneously showed increased AHR compared to wild-type mice (Fig. 2b). This suggests that ob/ob mice have innate AHR [12]. The relationship between leptin and airway physiology is U-shaped. Leptin enhances airway resistance, whereas loss of leptin can enhance airway resistance due to obesity. AHR is also observed in db/db mice (leptin receptor deficient obese) [12] and high-fat diet mice (leptin resistance) [27]. This suggests that obesity per se increases AHR in animal models. Low FRC and tidal volume induce a small airway caliber and result in increased AHR. Adipokines, including IL-6 and TNF-α, are reported to induce inflammation in obesity [28]. However, our data demonstrated that non-treated ob/ob mice showed no airway inflammation and mucus production histologically (Fig. 4a, b), supporting a previous report [12]. In addition, ob/ob mice showed less peri-bronchial fibrous tissue than wild-type mice in Masson’s trichrome staining (Fig. 4c). Leptin has been reported to induce interstitial fibrosis in some organs [29, 30]. Hypothetically, less fibrous tissue around the bronchi might have some influence on the collapsibility of bronchi and induce AHR in ob/ob mice. However, further investigations are required to verify this hypothesis.

Ob/ob mice showed significantly greater baseline Ers, G, and H than wild-type mice (Fig. 2a). As we conducted AHR measurements under closed-chest conditions, greater Ers, G, and H might be induced by mechanical factors of obesity per se. Abundant visceral adiposity can reduce lung volume and induce closure of the peripheral airway in ob/ob mice. Furthermore, Ers, G, and H were significantly increased in Leptin + IL-33-treated ob/ob mice (Fig. 2d), which was not observed in wild-type mice (Fig. 2c). Leptin + IL-33-treated ob/ob mice showed a greater response to methacholine than IL-33-treated or non-treated ob/ob mice (Fig. 2d). It can be reasonably explained that augmented airway inflammation and mucus secretion caused the response. In particular, we speculate that mucus secretion in Leptin + IL-33-treated ob/ob mice might easily obstruct peripheral airways and induce a greater response of Ers, G, and H to methacholine. As small airway closure is observed in obese subjects [31], mucus secretion might play a critical role in the decreased lung function and severe symptoms of obesity-associated asthma.

In wild-type mice, leptin administration prior to IL-33 induced AHR (Fig. 2c; Rrs). The addition of leptin to IL-33 induced a significant increase in BALF eotaxin and KC in wild-type mice (Fig. 3h, i). It has been suggested that augmented airway inflammation plays a role in the increased AHR in wild-type mice. Others also reported that the addition of OVA challenge [32] or IL-17A [33] to IL-33 enhanced AHR compared to IL-33 alone.

Concerning mucus production, our in vivo and in vitro studies suggested a synergetic effect of IL-33 and leptin. Leptin upregulates mucin expression via extracellular signal-regulated kinase (ERK1/2) and p38 pathways in vitro [21]. IL-33 induces mucin gene expression in human nasal epithelial cells [34]. IL-33 induces activation of NF-κB, ERK1/2, and p38 pathways [13]. IL-33 and leptin may induce mucin production in the airway through common signal transduction pathways, such as ERK1/2 and p38. The leptin receptor is expressed in NCI-H292 cells [21] and airway epithelial cells in asthma [17]. IL-33-treated basophils have higher levels of leptin receptors [35]. IL-33 might induce leptin receptor expression in the airway and enhance the effect of leptin treatment. Further investigation is required to clarify the interaction between IL-33 and leptin.

This study might be an ideal model for assessing the effects of leptin and obesity per se separately in an IL-33-induced asthma model. We confirmed that the maximal concentration of leptin in the serum was nearly the same in wild-type mice as in ob/ob mice 1 h after leptin injection (Fig. 1b). Moreover, during the experiment, body weight did not significantly change between the treatment groups (Fig. 1e). This enabled us to assess the effects of exogenous leptin treatment without the effects of body weight change.

We administered equal doses of IL-33 to ob/ob and wild-type mice. We preliminarily confirmed that the baseline levels of IL-33 in BALF were nearly the same. It has been reported that IL-33 and ST2 gene expression in adipose tissue is not different between genetically obese db/db mice and db/ + mice [36]. These data suggest that the equal doses of IL-33 might be adequate to our genetic mice. However, further studies are required to elucidate IL-33/ST2 expression in the lung tissue in our model. In addition, exogenous leptin administration is a classical technique to examine the effects of leptin in ob/ob mice [37]. Our results clearly proved the enhancement effect of IL-33.

Female mice were used in the present study. Obesity-associated asthma is known to be more prevalent in females than in males. Sood et al. reported that the association between leptin and asthma was stronger in women than in men [38]. Visceral fat leptin expression is significantly associated with AHR in women with asthma [39]. It is well known that obese women with asthma show severe airflow limitation, little eosinophilic inflammation, and steroid unresponsiveness [40, 41]. Uddén et al. showed that corticosteroids induce elevated serum leptin levels in women [42]. Increased body weight and leptin levels induce more AHR and worsen symptoms, especially in women. Further studies are required to clarify whether sex differences have any effect on the findings of our animal studies.

Based on the results of this experiment, we hypothesized that the mechanism of obesity-associated severe asthma is as follows. Increased body weight induces AHR due to mechanical factors, especially if the patients have innate AHR. When any insult occurs in the airway and IL-33 is released, IL-33 combined with leptin induces airway inflammation and goblet cell metaplasia. Even if the inflammation is mild (i.e., decreased type 2 inflammation), AHR may be enhanced as obesity-induced peripheral airway closure may be accelerated by mucus. Overall, the patients present with severe asthma. However, human obesity shows increased leptin levels, which differ from those in ob/ob mice. Even short-term high-fat diet treatment has been reported to induce leptin and AHR [43]. Therefore, further studies are required to confirm this hypothesis.

## Conclusions

In summary, we demonstrated that ob/ob mice show innate AHR without airway inflammation. IL-33 combined with leptin induced airway inflammation, goblet cell metaplasia, and enhanced AHR including peripheral airway closure. This is presumably accelerated by mucus in ob/ob mice. These results may explain some aspects of the pathogenesis of obesity-associated asthma. Furthermore, the control of mucus, including the target of IL-33 and leptin, may be a therapeutic strategy for obesity-associated asthma.

## Data Availability

The datasets used and/or analyzed during the current study are available from the corresponding author upon reasonable request.

## Abbreviations

AHR: Airway hyperresponsiveness
BALF: Bronchoalveolar lavage fluid
ELISA: Enzyme-linked immunosorbent assay
ERK1/2: Extracellular signal-regulated kinase 1/2
Ers: Elastance
FRC: Functional residual capacity
G: Tissue damping
H: Tissue elastance
HE: Hematoxylin–eosin
ILC2: Type-2 innate lymphocytes
KC: Keratinocyte-derived chemokine
OVA: Ovalbumin
PAS/AB: Periodic acid-Schiff/Alcian-blue
PBS: Phosphate-buffered saline
Rn: Newtonian resistance
Rrs: Resistance of the respiratory system
